# Lipid species dependent vesicles clustering caused by alpha-synuclein as revealed by single-vesicle imaging with total internal reflection fluorescence microscopy

**DOI:** 10.52601/bpr.2021.210020

**Published:** 2021-12-31

**Authors:** Chinta Mani Aryal, Owen Tyoe, Jiajie Diao

**Affiliations:** 1 Department of Cancer Biology, University of Cincinnati College of Medicine, Cincinnati, OH 45267, USA; 2 Department of Physics, University of Cincinnati, Cincinnati, OH 45221, USA

**Keywords:** Single molecule, Alpha-Synuclein, Lipids, Vesicles, Clustering, Total internal reflection fluorescence microscopy

## Abstract

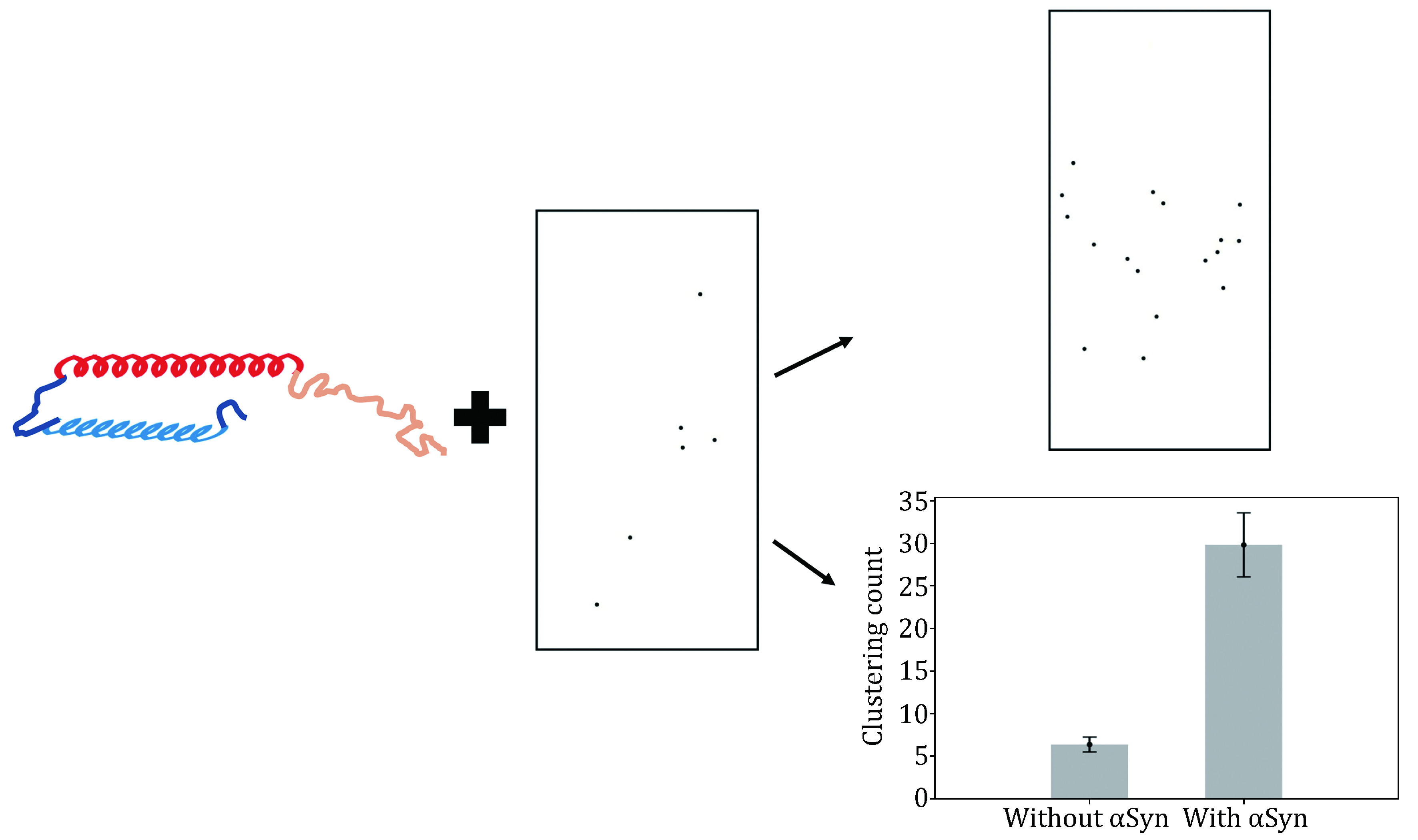

Single-molecule methods have been applied to study the mechanisms of many biophysical systems that occur on the nanometer scale. To probe the dynamics of such systems including vesicle docking, tethering, fusion, trafficking, protein-membrane interactions, *etc*., and to obtain reproducible experimental data; proper methodology and framework are crucial. Here, we address this need by developing a protocol for immobilization of vesicles composed of synthetic lipids and measurement using total internal reflection fluorescence (TIRF) microscopy. Furthermore, we demonstrate applications including vesicle clustering mediated by proteins such as alpha-Synuclein (αSyn) and the influence of external ions by using TIRF microscopy. Moreover, we use this method to quantify the dependence of lipid composition and charge on vesicle clustering mediated by αSyn which is based on the methods previously reported.

## INTRODUCTION

The series of events with synaptic vesicles in association with proteins are common in synaptic nerve terminals. For example, synaptic vesicles (SV) transportion, fusion, and recycling processes facilitate the release of neurotransmitters (Esposito *et al.* 2011). These overall processes take place in many steps such as the formation of cluster, docking, tethering, exocytosis and endocytosis cycle. This is, however, possible only after the mediation of cytosolic proteins (*e*.*g*., alpha-synuclein (αSyn)) (Murphy *et al.* 2000), membrane bound proteins *e*.*g*. soluble N-ethylmaleimide-sensitive factor attachment protein receptor (SNARE) (Bonifacino and Glick 2004) and other accessory proteins and ions. Specifically, αSyn w/o SNARE or ions plays a critical role for regulation in clustering (Cai *et al.* 2020; Diao *et al.* 2013), docking (Lai *et al.* 2014), tethering (Cai *et al.* 2019), fusion (Hu *et al.* 2019), and endo/exocytosis (Lautenschlager *et al.* 2017) by stabilizing the vesicles (Kaur and Lee 2021).

αSyn is a functional protein found predominantly in the cytosol of neurons with primarily high abundance in the presynaptic terminal (Stefanis 2012) and is associated with both physiology as well as pathology. Whereas the physiological role involves with synaptic vesicles recycling by interaction with the lipid membrane (Lautenschlager *et al.* 2017); the pathological role is relevant in several synucleinopathies such as dementia. Remarkably, its aggregation into the Lewy bodies becomes the hallmark of Parkinson’s disease (PD) (Spillantini *et al.* 1997). Indeed, the structural variation (Wang *et al.* 2016) or mutation (Polymeropoulos *et al.* 1997) or post-translational modifications (Wu *et al.* 2020; Bu *et al.* 2017) are causative for the functioning of αSyn from physiological function to the toxic condition. It is comprised of 140 amino acids (~14 kDa) and is known to be an intrinsically disordered protein. The structural basis shows that it can be divided into three main regions namely: N-terminus, non-amyloid-beta component (NAB) and C-terminus contributing distinct structural, and dynamical properties for the physiochemical regulation relevant to the content of amino acid sequences (Fusco *et al.* 2014). Of many functionalities of αSyn such as docking, tethering, fusion, endo/exo-cytosis are influenced by lipid species binding as mentioned earlier. Upon membrane binding, αSyn adopts the structural transition (alpha helix) (Pfefferkorn *et al.* 2012). Studies have shown that αSyn has a high tendency of binding only with the anionic lipids like phosphatidylserine (PS) (Middleton and Rhoades 2010), and is preferential to the small and highly curved membrane (Liu *et al.* 2021). However, needs a specific combination of polyunsaturated chains which provides the loose packing (Kubo *et al.* 2005). Other studies have shown that the presence of zwitterionic phospholipid particularly phosphatidylethanolamine (PE) causes the elevated binding (Jo *et al.* 2000), whereas Jianjun Pan *et al*. shows the inhibitory effect of PE in membrane remodeling (Pan *et al.* 2018). So, the distinct role of specific lipid’s head and chains for αSyn binding remain elusive.

Synaptic vesicles of size ~40 nm contain a significant amount of phospholipids with 12 mole% PS, 23 mole% PE, and several other lipids (cholesterol, spingomyline, phosphatidylinositol, hexylceramide, ceramide, *etc*.) covering ~50% surface, and is asymmetric in terms of lipid distribution (Takamori *et al.* 2006). Every lipid in synaptic vesicles has a specific role in association with the αSyn binding (Shvadchak *et al.* 2011). Biophysical studies of mimic synaptic vesicles (Crowe *et al.* 2017) or membranes mediated by αSyn *in vitro* (for detail refer to review Candace M Pfefferkorn *et al.* (Pfefferkorn *et al.* 2012)) have been studied by using different kinds of model vesicles and bilayer using variable lipids (Pan *et al.* 2018). So having an insight into how lipid species and their head or tail are affected by αSyn binding is important to understand the functionalities of synaptic vesicles recycling and pathogenicity caused by this protein.

**Figure 1:**
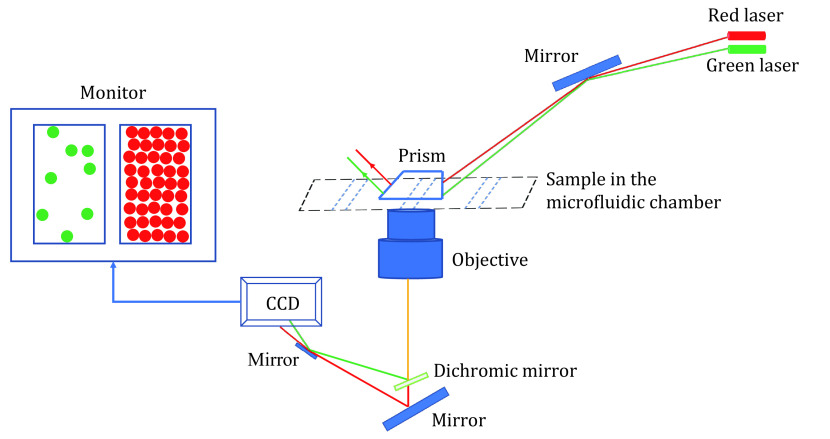
Schematic illustration of an imaging and detection system of the prism based TIRF

Several techniques have been employed to study the membrane association of αSyn such as nuclear magnetic resonance spectroscopy (NMR) (Eliezer *et al.* 2001), atomic force microscopy (AFM) and electron paramagnetic resonance spectroscopy (EPR) (Pan *et al.* 2018), computer simulation (West *et al.* 2016), neutron reflectometry (NR) (Hellstrand *et al.* 2013), electron microscopy (EM) (Li *et al.* 2019; Madine *et al.* 2009), *etc*. Here we study the interaction of αSyn and membrane for lipid specificity at the single-molecule level (Tian *et al.* 2019; Ferreon *et al.* 2009; Gong *et al.* 2016) which overcomes the limitation of ensemble average (Deniz *et al.* 2007) by using total internal reflection fluorescence microscopy (TIRFM or TIRF) (Lai *et al.* 2016; Hu *et al.* 2017; Du *et al.* 2020). TIRF is often known as evanescent wave or field microscopy providing a high contrast imaging of dynamics at or at the proximity of cellular membrane (Mattheyses *et al.* 2010). The physical basis of this technique is that it utilizes the evanescent field as an excitation field which decays exponentially with distance when the incoming bean passes through the medium with a high refractive index (usually glass) to a low refractive index (buffer or sample) at an angle above the critical angle (Fish 2009). This technique is often compared with epifluorescence microscopy. Unlike epifluorescence microscopy that fits for bulk imaging, it is capable to provide information about the process that happens within ~100 nm (Steyer and Almers 2001). So, utilizing this technique for selective excitation using two solid state lasers (green (532 nm)-Nd:YAG and red (640 nm)-HeNe) has been particularly useful to study the vesicles docking as induced by protein (Cai *et al.* 2019). Moreover, it has been established as a powerful technique to probe several modern processes in cell biology (Mattheyses *et al.* 2010). For instance, peptide–lipid interaction (Fox *et al.* 2009), actin nucleation and elongation (Jiang and Huang 2017), vesicles clustering as mediated by αSyn and regulated by calcium ions (Cai *et al.* 2020), translocation of signaling molecule (Tengholm *et al.* 2003), and docking and priming of vesicles (Becherer *et al.* 2007) have been successfully studied using TIRF. Figure 1 shows the schematic of measurement by prism based TIRF.

Small unilamellar vesicles (SUV) provide an excellent platform to monitor several biological phenomena *in vitro* (Hu *et al.* 2016; Aryal *et al.* 2020; Pan *et al.* 2017; Khadka *et al.* 2018, 2021). To monitor and quantify the lipid species dependent clustering synaptic mimicking SUV and effectiveness of the technique; in this paper, we report a protocol of small unilamellar vesicles immobilization followed by interaction with free vesicles (Diao *et al.* 2009) as mediated by synaptic protein αSyn as measured by TIRF. Specifically, vesicles of size ~45 nm with DiD labeled vesicles (DiIC18(5); DiD-vesicles onward) are immobilized and vesicles with DiI labelled (DiIC18(3); DiI-vesicles onward) are floating. The floating vesicles depending upon the lipid species it contains in the presence or absence of divalent cations show the distinct quantitative clustering in the presence of αSyn. Clustering as mediated by αSyn and the influence of divalent cations (Cai *et al.* 2019) particularly the concentration dependent non-linear role of Ca^2+^ has been already studied by Diao’s group (Cai *et al.* 2020). By following a similar protocol, it may be relevant to study the influence of lipid species such as PS at different concentration levels in the clustering of vesicles as mediated by αSyn and modulated Ca^2+^. Understanding the influence of particular lipid species on the protein induced process in a single vesicles level will provide the kinetic and dynamic insight into the mechanism of action at molecular level in cell membranes (Man *et al.* 2021). The method described here is particularly designed for those who wish to perform the imaging and analysis of protein mediated action at single-molecule level, but could be applied to study the other macromolecule-macromolecule interactions and endocytosis/exocytosis, budding, fusion/fission process, *etc*. A similar assay has been already discussed to study the conformational dynamics of αSyn during the interaction with the membrane (Ma *et al.* 2019) and SNARE (Sun *et al.* 2019) for its functional relevance.

## EXPERIMENTAL SECTION

### Materials

#### List of reagents

Reagents used in experiments are listed in Table 1.

**Table 1:** List of reagents

Name of reagents	Manufacturer	Country
KOH	Fisher Scientific	USA
Methanol	Fisher Scientific	USA
Acetic acid	Fisher Scientific	USA
Chloroform	Fisher Scientific	USA
Sodium bicarbonate	Fisher Scientific	USA
HEPES	Fisher Scientific	USA
m-PEG, poly(ethylene glycol) monomethyl ether	Laysan Bio, Inc.	USA
Biotin-PEG, biotinyl-polyethylene glycol	Laysan Bio, Inc.	USA
NeutrAvidin	Thermo Scientific	USA
DOPC, 1,2-dioleoyl-sn-glycero-3-phosphocholine	Polar Lipids, Inc.	USA
DOPE, 1,2-dioleoyl-sn-glycero-3-phosphoethanolamine	Polar Lipids, Inc.	USA
DOPS, 1,2-dioleoyl-sn-glycero-3-(phospho-l-serine)	Polar Lipids, Inc.	USA
Biotin-PE, 1,2-dioleoyl-sn-glycero-3-phosphoethanolamine-N-(biotinyl)	Polar Lipids, Inc.	USA
DiD, DiIC18(5)	Life Technologies Corporation	USA
DiI, DiIC18(3)	Life Technologies Corporation	USA
Amino silane, (3-(2-Aminoethylamino) propyl) trimethoxysilane	Sigma-Aldrich	USA
NaCl	Research Products, Inc.	USA
Acetone	VWR	USA
Ethanol	Decon Labs, Inc.	USA
alpha-synuclein	AnaSpec, Inc.	USA
Tris (hydroxymethyl) aminomethane	RPI	USA
Immersion oil	Olympus	Japan

#### List of equipment

Equipment that are used for experiment are tabulated in Table 2.

**Table 2:** List of equipment

Name of equipment	Manufacturer	Country
Aluminum foil	Great Value	USA
Razor blades	Personna	USA
Parafilm	Fisher Scientific	USA
Quartz slides with drilled holes (1 inch × 3 inch, 1 mm thick)	Finkenbeiner Inc.	USA
Coverslips (24 mm × 40 mm, rectangular)	VWR	USA
Epoxy (5 min)	Devcon	USA
Double-sided tape (~100 μm thick)	3M	USA
Propane torch	Fisher Scientific	USA
Forceps	Fisher Scientific	USA
Beakers	Fisher Scientific	USA
Ball container	Sigma-Aldrich	USA
Filter membrane (50 nm pores)	Polar Lipids, Inc.	USA
Filter support	Polar Lipids, Inc	USA
Syringes	Polar Lipids	USA
Glass tubes	Fisher Scientific	USA
Vortex mixture	Fisher Scientific	USA
Extruder	Polar Lipids, Inc	USA
640 nm laser	CrystaLaser LC	USA
532 nm laser	CrystaLaser LC	USA
Water-immersion objective lens	Olympus	Japan
Dichroic mirror	Chroma Technology Corp.	USA
Dual-band filter	Chroma Technology Corp.	USA
Pellin-broca prism (CVI laser)	CVI laser	USA
Electron-multiplying charge-coupled device (EMCCD) camera	Andor Technology Ltd.	UK

#### Table of % (mole) lipids in the vesicles

Lipids compositions for both DiD and DiI-vesicles are presented in Table 3.

**Table 3:** Lipid compositions (mol%) for DiD and DiI-vesicles

Lipid/Fluorescent molecules	DiD-vesicles			DiI-vesicles		
	30% PS	12% PS		30% PS	12% PS	No PS
DOPC	68.8	66.8		69	67	79
DOPE	−	20		−	20	20
DOPS	30	12		30	12	−
Biotine-PE	0.2	0.2		−	−	−
DiD	1	1		−	−	−
DiI	−	−		1	1	1

### Procedures

#### Research design

Based on the initial hypothesis that lipids species influence the αSyn binding (particularly PS, and PE), while the role of Ca^2+^ has been studied (Diao *et al.* 2009), it will change the ionic strength, suppressing the binding of αSyn at low concentration as a competitive binding. Acknowledging this idea, we experimentally validate the αSyn binding as caused by PE and PS, since both are presented in the synaptic vesicles. Furthermore, it has been reported that whereas phosphatidylcholine (PC) lipid plays little role in binding, PE has relatively higher; this study can be extended to understand the actual role of PE for αSyn interaction in a single molecule level. The experiment covers several tests for a given αSyn binding by identifying the optimized working condition satisfying the physiological relevance. Since experimental evidence shows the presence of anionic lipid enhance the binding of αSyn with vesicles, the project is being initialized with the extreme content of PS (Rhoades *et al.* 2006; Diao *et al.* 2009) for example using 30 mole% on both DiD and DiI-vesicles and or reproducing the earlier reported results from our lab on the effect of different concentrations of calcium ions. Below, we outline the methodology and preliminary results.

#### Sample preparation

1 Surface cleaning and PEGylation

(A) Surface cleaning

The pre-drilled quartz slides and the glass coverslips obtained from vendors must first be thoroughly cleaned. Here, we have described our standard cleaning procedure, but others have used slightly different protocols (Du *et al.* 2021; Lamichhane *et al.* 2010). Scrub and thoroughly rinse slides with ethanol (100%) and then with Milli-Q water. Since the presence of impurities on the surface increase background fluorescence, scrubbing is required for removing debris from previous experiments. Place slides and fresh coverslips in the clean and dry Coplin staining jars separately. Rinse with Milli-Q water. Sonicate with acetone for 20 min to remove any remaining organic material and non-specifically interacting debris. Again rinse with Milli-Q water three times. Fill jars with 1 mol/L KOH. Sonicate for 20 min followed by rinsing with Milli-Q water three times. Fill jars with methanol and sonicate for 20 min. Burn one side of each quartz slide for at least two mins using a propane torch, then rinse both slides and cover slips with fresh methanol, three times.

(B) Silanization

The next step is silanization which is achieved by aminosilanization with amino silane ((3-(2-aminoethylamino) propyl) trimethoxysilane (APTS)). In aminosilanization reaction, methanol is used as a solvent and acetic acid as a catalyst (Chandradoss *et al.* 2014). Mix 100 mL methanol, 5 mL acetic acid and 1 mL APTS reagent in a clean, dry beaker. Pour into the jars and make sure it fully covers slides and coverslips. Incubate for 10 min. Sonicate for 1 min and again incubate for 10 min. Rinse slides and coverslips with methanol, and then at least three times with Milli-Q water. Completely dry the slides and coverslips with blowing air. Note that, amino silane solution should be freshly prepared.

(C) PEGylation

Polyethylene glycol (PEG) is commonly used for surface passivation to prevent non-specific interactions with protein, lipids (Ha and Joo 2002; Joo and Ha 2012a) or vesicles (Wang *et al.* 2019). This is achieved by adding 100 µL of freshly prepared reaction solution (120 mg of mPEG, 4 mg of biotin–PEG, and 700 µL of 0.1 mol/L sodium bicarbonate solution) onto the imaging surface of the slide. Each slide was covered by one clean coverslip to be a set, which was incubated overnight and then rinsed with Milli-Q water, dried, used immediately or stored at 20 ^o^C for further use.

(D) Microfluidic chamber assembly

Prepare 4–8 mm wide and ~150 μm deep flow channels. For this place a quartz slide on a flat surface with the PEGylated side facing up. Make a channel on the PEGylated surface by putting double-sided tape over the quartz slide in such a way that the holes are positioned at the center of the channel. Place a coverslip facing the PEGylated surface at the top of taped quartz slide. Remove the extra tape by using a razor blade. Seal the chamber by pressing the coverslip over the area where double-sided tapes are placed using properly mixed epoxy (5 min) and wait at least 20 min for the epoxy to set.

2 Vesicles preparation

**Figure 2:**
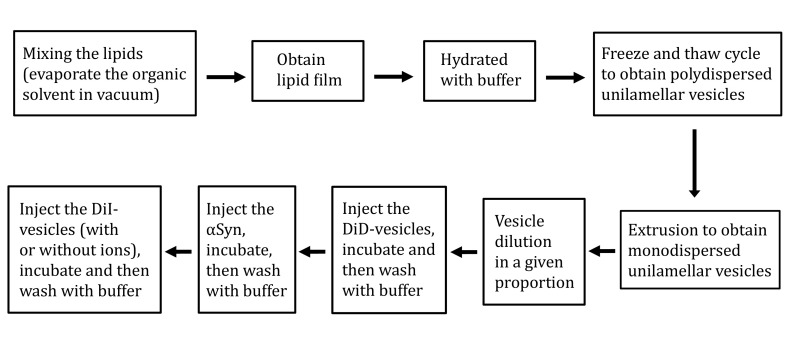
Flow diagram to show the steps of vesicles and sample preparation

SUV were prepared by using the extrusion method discussed elsewhere (Aryal *et al.* 2020). Briefly; Mix the lipids in the appropriate ratio (Table 3) from stock kept at –20 ^o^C in a glass tube. For each consecutive lipid pipetation, the syring should be cleaned at least five times. Vacuum the mixture for 4 h to remove the organic solvents (chloroform/ethanol). Rehydrate the obtained dry film with HEPES (25 mmol/L HEPES, 100 mmol/L NaCl, pH 7.4 (with NaOH)) buffer. Vertex and sonicate the suspension until the film is fully dissolved. Repeat seven cycles of freezing and thawing by submerging into the water at 50 ^o^C and introducing into the dry ice (–79 ^o^C) alternatively to obtain the polydispersed unilamellar vesicles solution. Finally, obtain the monodispersed vesicles (size ~50 nm) by extruding the solution (21 times) through the 50 nm polycarbonate membrane. Use the obtained vesicles immediately or keep them at –80 ^o^C for future use. Note that, chloroform is a carcinogenic agent, lipid mixing is advised to be conducted under a fume hood. Vesicles preparation steps are summarized in Fig. 2.

3 Vesicles immobilization and slide preparation

For the measurement by using TIRF, inject 40 µL (two shots of 20 µL) of 0.1 mg/mL NeutrAvidin (Thermo Scientific, 0.1 mg/mL in 10 mmol/L Tris-HCL, pH 7.5, 50 mmol/L NaCl) per channel and incubate for 15 min. This will create a binding surface for biotin labeled samples (DiD-vesicles). Inject the 100 µL of DiD-vesicles with appropriate dilution and incubate for 30 min. This is the basis framework for many types of experiments, but for us, we are interested in vesicle clustering and the interaction with αSyn. Inject 50 µL of αSyn (appropriate concentration) and incubate for 30 min. Inject 100 µL DiI-vesicles with appropriate dilution and incubate for 30 min. The channel without αSyn is considered as the control channel. Note, in each consecutive step, HEPES buffer exchange is required at least five times (200 µL each time) to remove unbound vesicles/protein. Thus, prepared slides were imaged using TIRF under identical intensity.

DiD-vesicles which contain biotin–PE moiety bounds to the neutravidin and hence get immobilized. Since an optimized number of DiD-vesicles provides the homogenous distribution, it can be visualized using the red laser under TIRF. After the confirmation of DiD-vesicles coverage, the protein was injected for the affected channel whereas only buffer was injected in the control channel. Control and affected channel should be prepared within a slide leaving the consecutive channel without any sample to save it if there is any tape to tape leakage, and for better batch to batch comparison. Replication was also done within the same slide for the comparison. Since αSyn has a definite binding ability, the optimal DiI-vesicle concentration depends upon protein. The imaging sequence is tabulated in Table 4.

**Table 4:** Imaging sequence

Action	Reason
Focus	Initial focusing is preferred with green laser because of the low photobleaching effect
DiD-vesicles with red laser	To confirm the background/substrate vesicles if they are distributed homogenously
DiD-vesicles with green laser	To check if there are vesicles in the background that can be probed by green (ideally zero)
DiI-vesicles w/o protein or ions with green laser	To examine the clustering/tethering of DiI-vesicles on top of DiD-vesicles driven or not by protein or ions
Whole sample set with red laser	To identify if DiD-vesicles are still there as a substrate; confirmatory test to assure the DiI-vesicles tethering is on the top of DiD-vesicles (but not due to in-specific binding)

After the experiment, the slides can be reused. For recycling, soak the slide in acetone overnight to soften any residue of epoxy or tape from the previous experiment. Use a razor blade to scrape off the residue. Rinse the slides several times with Milli-Q water and dry them.

4 Optimization of working condition

There are many variables to consider to obtain consistent and reproducible results and optimization relies on fixing many of the variables to be as uniform as possible. Beyond the laser alignment, one must optimize the surface density of bound substrate vesicles (DiD-vesicles) *e*.*g*., full coverage, homogenous and isotropic distribution in the channel, number (ratio) of DiD and DiI-vesicles for proper concentration of αSyn (pmol/L to µmol/L) binding (Diao *et al.* 2012), choice of background (high or low), time of incubation (*e*.*g*., few minutes to many hours), temperature (about 0 ^o^C or room temperature (RT) or 37 ^o^C), DiD or DiI-vesicles as substrate, mole% of DiD and DiI fluorescent in a given composition and any surface imperfections on the quartz surface of oil-prism interface which results in the formation of diffraction patterns in the images which in turn make them useless. It is to be noted that, the vesicles count differs from channel to channel, sample to sample and batch to batch, so direct comparison of the count may not be possible.

5 Buffer preparation

PEG buffer: this is prepared freshly for immediate use. It contains 0.1 mol/L sodium bicarbonate (pH 8.5).

HEPES buffer: this can be used for up to one month if stored at 4^ o^C. It contains 25 mmol/L HEPES and 100 mmol/L NaCl (pH 7.4 with NaOH).

T50 buffer: this can be stored at 4 ^o^C to be used for a month. It contains 10 mmol/L Tris-HCl and 50 mmol/L NaCl (pH 8.0 with NaOH).

#### TIRF imaging

**Figure 3:**
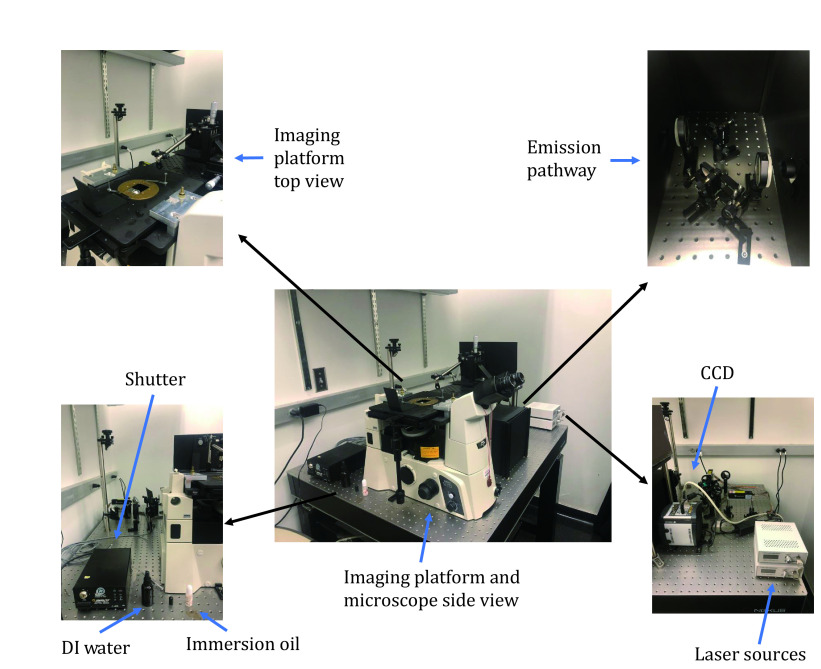
Photograph of the different parts of the TIRF system in the lab

The sample is mounted in the following sequence for the imaging: DI water drop at the top of objective, sample coverslip down in contact with DI water, immersion oil on the surface of quartz slide, and finally the prism at the top of the oil. The prism is held in place with a frame and screw. The photograph of the TIRF system used for this experiment is as shown in Fig. 3.

For reproducibility, at least ten different randomly selected sites were scanned for each sample flow channel and at least three channels a day were scanned. The experiment was conducted at least three different days with freshly prepared vesicles.

#### Image processing and quantification

Fluorescence signals or images, as well as real time recording, can be taken and analyzed using separate software, but we do both using the custom-built software obtained from Dr. Taekjip Ha’s group (John’s Hopkins University, USA). Statistical results and plots are typically obtained in external programs, such as Excel, MATLAB, Python, Mathematica, *etc*. Protein induced changes are analyzed by using the student *t*-test. If *p* < 0.05, the change is considered statistically significant.

#### Imaging sequences

The following sequence (Table 4) was implemented in the imaging process.

#### Imaging troubleshoot

Here are some issues experienced and solutions associated with the imaging by TIRF and samples during the experiments (Table 5). For more details, please refer to our previous publication (Diao *et al.* 2012).

**Table 5:** Issues and troubleshoot

Issue	Reason	Solution
Parabolic shaped photobleaching covering at the reasonable field of view with high intensity	Improper focus with high intensity, high concentration of vesicles	Play with the focus knob gently. There are photobleaching in either direction, but there is a short window where the focus can be made, and lower the intensity, if not fixed change the vesicles concentration
Bleeding of vesicles in irregular pattern through-out the region	Tunneling of vesicles inside the tape; causing the unusual maximum intensity while playing with X-Y knob	At this point, the actual beam might be hitting off-center the objective which misleads with pattern or number in the screen. Reposition the beam is required
False center	Sample can be seen around the actual center as if it is focused but goes away while moving the position	Identify the proper center by playing with X-Y knob which gives the high intensity number
False focus	Sample can be visualized even if the beam is at the other edge of aperture showing the fuzzy focus	Realign the beam by taking the slide out in such a way that the initial beam (without slide and prism) will be at the inner side of the microscope’s aperture
Unstable imaging	Unlikely to focus the sample properly	Need to wash a sample properly with HEPES
Bad sample	Whiteness in tape caused by channel to channel sample leakage	Whiteness at the edge of tape may be ok, but if the tape separating the flow channel turns out to be completely white, it is likely caused by the tape to tape leakage of neutravidin or sample itself. Skip conjugate channel to load the sample or prepare a new slide

## EXPECTED RESULTS

**Figure 4:**
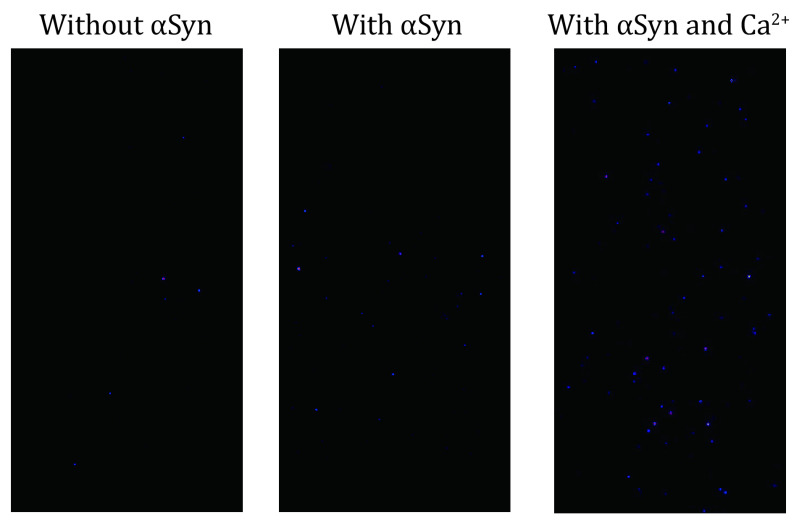
Representative images of DiI-vesicle clustering before and after protein and/or ion addition under the excitation of green laser

**Figure 5:**
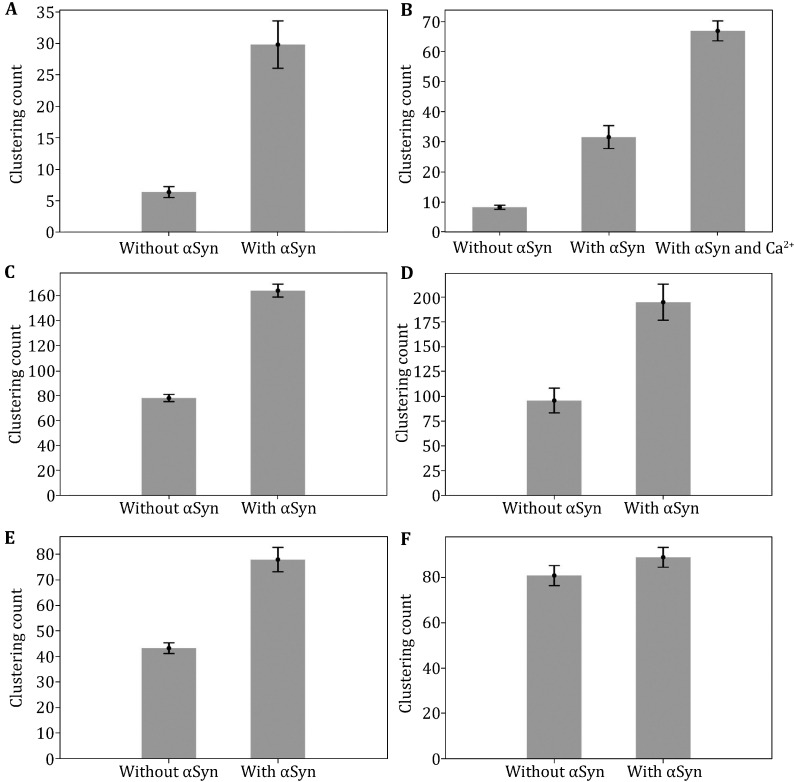
Statistic results on DiI-vesicle clustering count induced by αSyn and/or ion (mean ± SEM). **A** 12% PS containing DiI-vesicles on 12% PS containing DiD-vesicles (2 µmol/L αSyn; *p* < 0.001). **B** 12% PS DiI-vesicles with 10 mmol/L Ca^2+^ ion on 12% PS DiD-vesicles (5 µmol/L αSyn; *p* < 0.001 with respect to both controls, *i*.*e*., “without αSyn” and “with αSyn”). **C** 30% PS DiI-vesicles on 30% PS DiD-vesicles (20 nmol/L αSyn; *p* < 0.001). **D** 12% PS DiI-vesicles on 30% PS DiD-vesicles (20 nmol/L αSyn; *p* < 0.001). **E** No PS DiI-vesicles on 30% PS DiD-vesicles (20 nmol/L αSyn; *p* < 0.001); **F** No PS DiI-vesicles on 12% PS DiD-vesicles (2 µmol/L αSyn; difference is statistically insignificant). All the incubation steps and imaging were conducted at room temperature. The significance was calculated by Student’s* t*-test

Figure 4 shows the representative images of clustering of vesicles as an effect of αSyn or ion binding under the excitation of green laser. We quantify the observation by measuring the number of DiI-vesicles as a result of αSyn binding as a function of lipid head charge and ion concentration. As the measurement is done with various kinds of samples in the optimization process with different αSyn concentrations, we present the representative data here. Figure 5A shows the change in the clustering count of DiI-vesicles on the top of DiD-vesicles (both contain 12 mol% PS) as the effect of αSyn. Where-as an enhanced count can be observed for the same set of vesicles with the calcium ion as compared to both “without αSyn” and “with αSyn” as shown in Fig. 5B. Experiments were also performed in high background. Figure 5C and 5D show the significantly increased count as a result of αSyn binding in 30 mol% PS containing DiI-vesicles and the 12 mol% PS DiI-vesicles respectively, where, 30 mol% PS containing DiD-vesicle was the substrate. Furthermore, No PS DiI-vesicles (on 30 mol% PS DiD-vesicle substrate) show less enhanced count even in the presence of αSyn (Fig. 5E). However, No PS DiI-vesicles on 12 mol% PS DiD-vesicles show a pretty much similar count as background (Fig. 5F). With these preliminary results, it can be said that; αSyn shows a strong binding affinity with the lipids having charged head specifically PS, and highly enhanced in the presence of a higher concentration of calcium ion is in good agreement with the previous finding (Cai *et al.* 2020).

## SUMMARY

Surface passivation and immobilization strategy and measurement by TRIF have been widely accepted technique to study the several biophysical interactions in a single molecule level (Joo and Ha 2012b; Lamichhane *et al.* 2010). This technique and protocol can be generalized in different ways including to study the interacting behavior of protein and lipid in real time and to probe the complex system of proteins and lipids relevant to physiological conditions by using multi-color fluorophore/excitation. Furthermore, TIRF coupled with other high-resolution techniques and single level spectroscopies to probe molecular mechanisms and dynamics in the qualitative as well as quantitative manner. Here, we have described the detailed protocol in the quantification of the role of lipid’s charge or ion on clustering behavior caused by αSyn by using TIRF aiming to extend our study in αSyn’s binding with several other lipids that are physiologically relevant for synaptic vesicles. Since this assay has been already proven to be useful in elucidating the conformational dynamics of a protein in protein–membrane interaction and RNA/DNA–protein interaction in conjugation with single-molecule forster resonance energy transfer, it can be applied to study other protein–lipids, protein–protein, and protein–ion interactions. One immediate and potential application of this protocol could be to distinguish between the forces such as charges versus hydrophobicity and imperfection that govern the protein interaction with lipids species. Furthermore, this protocol could be extended in the *in vitro* biophysical studies of protein reconstituted system of vesicles (*e*.*g*., segment of SNARE) for their relevancy of docking, fusion *etc*. with the membrane.

## Conflict of interest

Chinta Mani Aryal, Owen Tyoe and Jiajie Diao declare that they have no conflict of interest.
